# Complex I reductions in the nucleus basalis of Meynert in Lewy body dementia: the role of Lewy bodies

**DOI:** 10.1186/s40478-020-00985-8

**Published:** 2020-07-09

**Authors:** Christopher Hatton, Amy Reeve, Nichola Zoe Lax, Alasdair Blain, Yi Shiau Ng, Omar El-Agnaf, Johannes Attems, John-Paul Taylor, Doug Turnbull, Daniel Erskine

**Affiliations:** 1 Wellcome Centre for Mitochondrial Research, Newcastle University, Newcastle upon Tyne, UK; 2 Translational and Clinical Research Institute, Newcastle University, Newcastle upon Tyne, UK; 3 Neurological Disorders Research Center, Qatar Biomedical Research Institute (QBRI), Hamad bin Khalifa University (HBKU), Qatar Foundation, Ar-Rayyan, Qatar

**Keywords:** Acetylcholine, Lewy body dementia, Alpha-synuclein, Mitochondria

## Abstract

Neurons of the nucleus basalis of Meynert (nbM) are vulnerable to Lewy body formation and neuronal loss, which is thought to underlie cognitive dysfunction in Lewy body dementia (LBD). There is continued debate about whether Lewy bodies exert a neurodegenerative effect by affecting mitochondria, or whether they represent a protective mechanism. Therefore, the present study sought to determine whether the nbM is subject to mitochondrial dysfunctional in LBD and the association of Lewy body formation with such changes. *Post-mortem* nbM tissue was stained for Complex I or IV and quantitated relative to porin with immunofluorescence using confocal microscopy of individual cells from LBD (303 neurons, 8 cases), control (362 neurons, 8 cases) and asymptomatic incidental LBD (iLBD) cases (99 neurons, 2 cases). Additionally, α-synuclein, tau and amyloid-β pathology were analysed using quantitative immunohistochemistry, and respiratory chain markers were compared in cells with Lewy bodies (*N* = 134) and unaffected cells (*N* = 272). The expression of Complex I normalised to mitochondrial mass was significantly lower in LBD compared to control and iLBD cases and this was unrelated to local neuropathological burdens but trended toward a relationship with neuronal loss. Furthermore, Complex I expression was higher in cells with Lewy bodies compared to adjacent cells without α-synuclein aggregates. These findings suggest that Complex I deficits in the nbM occur in symptomatic LBD cases and may relate to neuronal loss, but that contrary to the view that Lewy body formation underlies neuronal dysfunction and damage in LBD, Lewy bodies are associated with higher Complex I expression than neurons without Lewy bodies. One could speculate that Lewy bodies may provide a mechanism to encapsulate damaged mitochondria and/or α-synuclein oligomers, thus protecting neurons from their cytotoxic effects.

## Introduction

Lewy body dementia (LBD) is the second most common form of late-life neurodegenerative dementia after Alzheimer’s disease (AD), accounting for up to 20% of all cases [15]. LBD is a collective term for dementia with Lewy bodies (DLB) and Parkinson’s disease dementia (PDD), which are clinically distinguished on the basis of the temporal onset of symptoms, with cognitive symptoms preceding or occurring concurrently with motor symptoms in DLB whereas motor symptoms are a presenting feature of PDD [24]. DLB is marked by particularly accelerated cognitive decline, even compared to AD [16], yet there is an absence of therapies that can offer anything but modest symptomatic treatment [8]. Therefore, there is a pressing need to better understand the mechanisms that underlie cognitive impairment in LBD in the hope that these may contribute to the development of disease-modifying therapies.

The accumulation of the protein α-synuclein into well-defined intracellular aggregates termed Lewy bodies is the characteristic neuropathological feature of LBD [27]. However, it should be noted that AD pathology, consisting of hyper-phosphorylated tau and amyloid-β, is typically a concomitant feature at levels intermediate between normal ageing and AD [24]. Although Lewy bodies are necessary for a neuropathological diagnosis of LBD, their relationship with clinical symptoms is controversial, with studies showing conflicting findings relating Lewy body burden to important clinical or pathological variables [13, 14, 28, 43]. Despite the controversy surrounding the role of Lewy bodies in neurodegeneration in LBD, inhibition of α-synuclein aggregation into Lewy bodies remains a major goal of candidate therapeutics [17, 33, 46].

It is thought that the aggregation of α-synuclein from an unstructured monomer into oligomeric and/or fibrillar aggregates characterised by β-sheet rich structures mediates the cytotoxicity of α-synuclein through either a loss of normal protein function or a gain of toxic function [27]. α-Synuclein is known to interact with mitochondria in vitro, where it inhibits Complex I of the mitochondrial respiratory chain, and initiates a cascade of oxidation events culminating in opening of the permeability transition pore, mitochondrial swelling and cell death [20]. Consistent with mitochondrial interactions driving cellular dysfunction and death in LBD, *post-mortem* studies have consistently reported Complex I deficiency in neurons in the substantia nigra, a region whose degeneration is thought to underlie parkinsonian symptoms in LBD [34, 36]. Other studies have reported Complex I deficiency in other brain regions, including those without Lewy bodies [12].

The nucleus basalis of Meynert (nbM) is a diffuse nucleus located in the basal forebrain. It is the largest of four cell groups in the basal forebrain (Ch1 – Ch4), which provide cholinergic innervation to widespread brain regions [25, 26], with the nbM (Ch4) providing cholinergic efferents to the entire cerebral cortex [26]. Neuronal loss and Lewy body pathology has long been recognized in the nbM and is associated with cognitive impairment in a number of clinical dementias, including Alzheimer’s disease and LBD [30, 44, 45]. The cells of the nbM are part of a neuromodulatory network with long axons and diffuse arborisations and are, thus, highly energy demanding [39]. Given the high-energy demands of nbM cells, one could speculate they may be susceptible to neurodegeneration secondary to mitochondrial dysfunction.

The present study sought to determine whether the nbM is subject to reductions in complexes of the mitochondrial respiratory chain in LBD, and whether such changes are associated with the burden of Lewy body pathology. To better understand the relationship between mitochondrial respiratory chain changes and α-synuclein pathology we included participants with incidental Lewy body disease (iLBD), individuals with Lewy body pathology in the nbM but an absence of cognitive or motor symptoms. We used the nbM as an exemplar region as it is subject to severe Lewy body pathology and cell-loss that is thought to underlie cognitive symptoms in LBD. Unlike previous studies, which have relied on light microscopic analysis of sections stained with single antibodies, we have employed the novel approach of quadruple immunofluorescence of individual cells with confocal microscopy [18]. This approach enables respiratory chain markers to be normalised to the total mass of mitochondria per cell, allowing precise determination of specific deficits irrespective of differences in mitochondrial mass.

## Methods

### Case selection

Cases were obtained from Newcastle Brain Tissue Resource based on tissue availability (Table 1). We selected a sub-set of LBD cases on the basis of a neuropathological diagnosis of LBD and an absence of concomitant pathologies (Braak tau stage <IV, absence of significant vascular pathology). Control cases were selected based on documented evidence of intact cognition proximal to death and an absence of significant age-associated pathology (Braak tau stage <II, absence of Lewy body and vascular pathology). iLBD cases were only included if they had clear evidence, from carer report or clinical evaluation, of intact cognition immediately prior to death and Lewy body pathology stage higher than 4, the stage at which the nbM is affected [5].

**Table 1:** Demographic data from the present cohort. “NFT Braak” refers to neurofibrillary tau pathology stage, “Thal phase” refers to amyloid-β phase, and “LB Braak” refers to Lewy body pathology stage

Case ID	Age	Sex	NFT Braak	Thal phase	LB Braak	Number of cells analysed
Control 1	80	M	I	1	0	46
Control 2	54	F	II	5	0	50
Control 3	59	M	I	3	0	49
Control 4	81	F	I	3	0	36
Control 5	63	M	I	3	0	49
Control 6	97	M	II	2	0	35
Control 7	92	F	I	2	0	50
Control 8	96	F	I	2	0	47
iLBD 1	92	F	II	5	5	49
iLBD 2	98	F	II	2	5	50
LBD 1	90	M	III	5	6	35
LBD 2	87	F	II	5	6	30
LBD 3	76	M	II	5	5	20
LBD 4	78	M	III	1	6	51
LBD 5	74	M	III	5	6	38
LBD 6	82	M	I	1	6	30
LBD 7	92	M	III	2	6	50
LBD 8	81	M	III	4	6	49

### Post-mortem tissue preparation

All brain tissue was obtained from Newcastle Brain Tissue Resource (NBTR), a UK Human Tissue Authority–approved research tissue depository, and ethical approval was granted by Newcastle University ethics board and the Joint Ethics Committee of Newcastle and North Tyneside Health Authority (ref: 08/H0906/136). All cases and controls had consented to the use of their brain tissue for research purposes. At autopsy, the right hemisphere was fixed in 10% formalin for 6 weeks prior to paraffin wax embedding. Irrespective of clinical diagnosis, all brains underwent neuropathological assessment [4, 5, 24, 40], and clinical and pathological data were collated to establish a consensus clinico-pathological diagnosis.

### Neuropathology

Seven micrometers sections containing nbM tissue was stained for ChAT, α-synuclein, amyloid-β and tau using previously reported methods [10]. α-Synuclein immunohistochemistry employed the combined approach of EDTA pH 8 and formic acid antigen retrieval and immunostaining with the KM51 antibody (1:250). Antigen retrieval for amyloid-β utilised formic acid and the 4G8 antibody (1:10,000), and tau used citrate pH 6 and the AT8 antibody (1:1000). Antibody immunopositivity was visualised using Menarini MenaPath X-Cell Linked Plus HRP detection systems, as per manufacturer’s instructions. A region of interest was drawn around the neurons of the nbM and immunopositivity determined using bespoke thresholds, with percentage area immunoreactive calculated using Image-Pro software (Media Cybernetics, Japan).

### Immunofluorescent staining of brain tissue

**Fig. 1:**
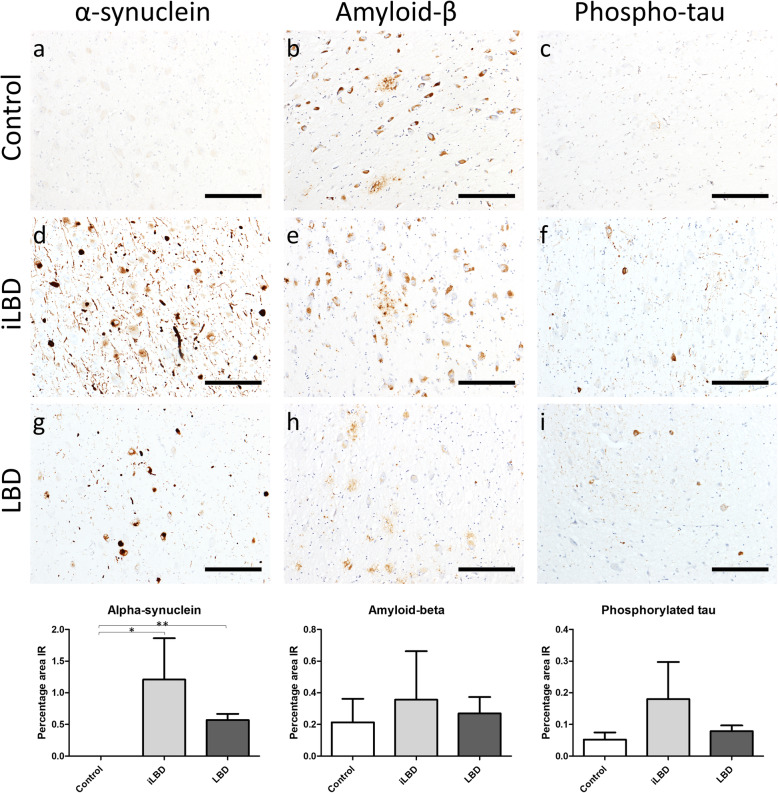
Representative images of neurodegenerative pathologies in control (Control 5; **a**-**c**), iLBD (iLBD 1; **d**-**f**) and LBD (LBD 2; **g**-**i**) nbM. Only α-synuclein burden differed across groups, where it was higher in iLBD and LBD compared to control. There was no significant difference in α-synuclein levels between iLBD and LBD. *p < 0.05, **p < 0.01

To evaluate changes to the mitochondrial respiratory chain in individual nbM cells, we employed a modified version of a previous protocol [18]. Firstly, to determine whether changes to mitochondrial respiratory chain subunits are found in the nbM in LBD, we co-stained nbM sections with the cholinergic neuron marker ChAT (28), a subunit of Complex I (NDUFB8; NADH dehydrogenase [ubiquinone] 1 beta subcomplex unit 8), a subunit of Complex IV (COX4; cytochrome oxidase subunit 4) and a marker of mitochondrial mass (porin/VDAC1; Fig. 1). Complex I and IV were evaluated as they have been previously been reported to be deficient in both mitochondrial disease [18] and Parkinson’s disease substantia nigra [34, 36].

For our second experiment to compare expression of mitochondria respiratory chain subunits in nbM neurons with Lewy bodies, compared to those without Lewy bodies, we replaced COX4 with the α-synuclein fibril marker, Syn-F2 [41]. This antibody was chosen as its isotype is mouse IgG2a, which is identical to that of COX4, thus enabling it to be easily multiplexed with the other primary antibodies.

The immunofluorescence protocol involved deparaffinisation and rehydration of sections followed by antigen retrieval in 1 mmol EDTA pH 8 in a pressure cooker for 40 min. Samples were blocked in 10% normal goat serum suspended in tris-buffered saline with 1% Tween-20 (TBST) for 1 h at room temperature. Antibodies against NDUFB8 (Abcam ab110242, 1:100), COX4 (Abcam ab110261, 1:100), porin/VDAC1 (Abcam ab14734, 1:200) and ChAT (Sigma HPA048547, 1:100) were suspended in 10% normal goat serum in TBST overnight at 4 °C. In the second experiment, Syn-F2 (El-Agnaf laboratory, Qatar; 1:1000) replaced COX4. After washing with TBST, secondary antibodies (Alexa Fluor 405-conjugated anti-rabbit antibody, 1:100; Alexa Fluor 546-conjugated IgG1 anti-mouse antibody, 1:100; Alexa Fluor 647-conjugated IgG2a anti-mouse antibody, 1:100; Alexa Fluor 488- conjugated IgG2b anti-mouse antibody, 1:100) were incubated for 1 h at room temperature and washed off with TBST. Autofluorescence was quenched by application of 3% Sudan Black B for 10 min at room temperature before slides were air-dried and mounted in ProLong Gold antifade.

### Confocal microscopy of single nbM neurons

Immunofluorescence was conducted using a Leica SP8 confocal microscope. The nbM was analysed at the level of the amygdala based on the Newcastle Brain Tissue Resource dissection protocol, and located based on its location on the ventral aspect of the globus pallidus and by the expression of ChAT in its distinctive large neurons. All neurons were imaged at 63x oil immersion lens and laser intensity settings were kept constant for every case. The zoom and z stack functions were used to image individual ChAT-positive neurons in three dimensions across all evaluated channels. Z-stacked images were obtained to avoid the introduction of bias by imaging of single two-dimensional points that would be arbitrarily determined by the operator. Three-dimensional images were compressed into a single two-dimensional image and saved in greyscale to avoid bias in the analysis of intensity by false colouring. We aimed to obtain 50 neurons per case or, if fewer neurons were present, every cholinergic neuron present in the region of interest on the section.

For densitometric analysis of the target proteins, ImageJ software (U.S. National Institutes of Health, Bethesda, MD, USA) was used and images from the four channels were imported as an image sequence. The background signal for each antibody was obtained by analysing staining intensity in single immunonegative regions proximal to the region of interest. This was measured every five cells, from which a mean background measure was calculated per slide, and subtracted from each measurement. Cells were outlined manually based on ChAT and/or porin immunostaining (porin was used when cells had low levels of ChAT meaning cell outlines were less clear) and mean optical densities (OD) from all channels were obtained using the “analyse stack” function in ImageJ, giving an optical density (OD) value for each channel in every cell. Background corrected OD values for individual cells were derived by subtracting the mean background signal from each data point for each cell.

### Analysis of respiratory chain marker expression in individual neurons

Analysis and comparison of ChAT and porin were conducted based on staining intensity irrespective of cell size to ensure that optical density measures, which combine intensity and area stained, would not bias analysis in favour of larger cells and, thus, give lower expression levels in atrophic cells. Such analysis could show decreases in ChAT secondary to cellular atrophy, not due to reduced expression in cells.

Background-corrected OD values of NDUFB8 and COX4 were normalised to background-correct porin OD values and log-transformed to normalise data. Z-scores per cell were determined relative to the normal population (i.e. all ChAT-positive neurons in the control group, *N* = 362) and compared on the group level by pooling all cells within groups.

### Comparison of complex I expression in cells with and without Lewy bodies

We further investigated Complex I expression based on its higher magnitude of deficiency in nbM neurons and its relationship to α-synuclein aggregation in vitro [20, 23]. Therefore, we compared cells without α-synuclein aggregates to those with either Lewy bodies, or to those with diffuse aggregates, using the same methods as employed to determine Complex I expression previously. In this analysis, we evaluated all cells per nbM, which also gave the total number of cholinergic neurons per LBD or iLBD case. We also quantified the area occupied by the Lewy body and subtracted this from each channel to determine whether accumulated mitochondria within Lewy bodies was artefactually altering expression. For this aspect of the study, laser settings had to be optimised due to use of a different batch of Sudan Black B that subtly altered staining intensity, thus the results from this part of the study could not reliably be converted to z scores using control data from the first part of the study, and thus ratios were used instead.

### Statistical analysis

All data were evaluated to determine whether they adhered to a normal distribution using visual inspection of Q-Q plots and Shapiro-Wilk tests, and heterogeneity of variance was evaluated using Levene’s test of normality of error variances. Complex I and IV expression from individual cells were analysed on the group level using a mixed-effect model, with the individual cases included as random effects. This analysis enabled comparison of all cells between groups, whilst taking into account that pooled observations from different cases cannot be considered independent observations. ANOVA or Kruskal-Wallis tests followed by appropriate post-hoc tests were used as appropriate, and parametric or non-parametric correlational analyses used to evaluate relationships, as appropriate. All analyses were performed using GraphPad Prism 8 software except the mixed-effects model which was performed in R.

## Results

### Demographics and neuropathology

There were no significant differences in the proportions of men/women across groups (χ^2^ = 5.90, df = 2, *p* = 0.052); however, a trend was observed towards a higher proportion of men in the LBD group, consistent with previous studies showing LBD to be more common in men [35], and the iLBD group was exclusively female. No significant differences were observed in the age of individuals across groups (K-W χ^2^ = 3.362, df = 2, *p* = 0.186). All demographic data are summarised in Table 1.

Analysis of α-synuclein, amyloid-β and tau demonstrated that only α-synuclein differed on the group level, and this was in controls compared to LBD cases (*p* < 0.01) and iLBD cases (*p* < 0.05). There was no difference between LBD and iLBD on any measure (Fig. 1).

### Complex I is decreased relative to porin in LBD but not iLBD

**Fig. 2:**
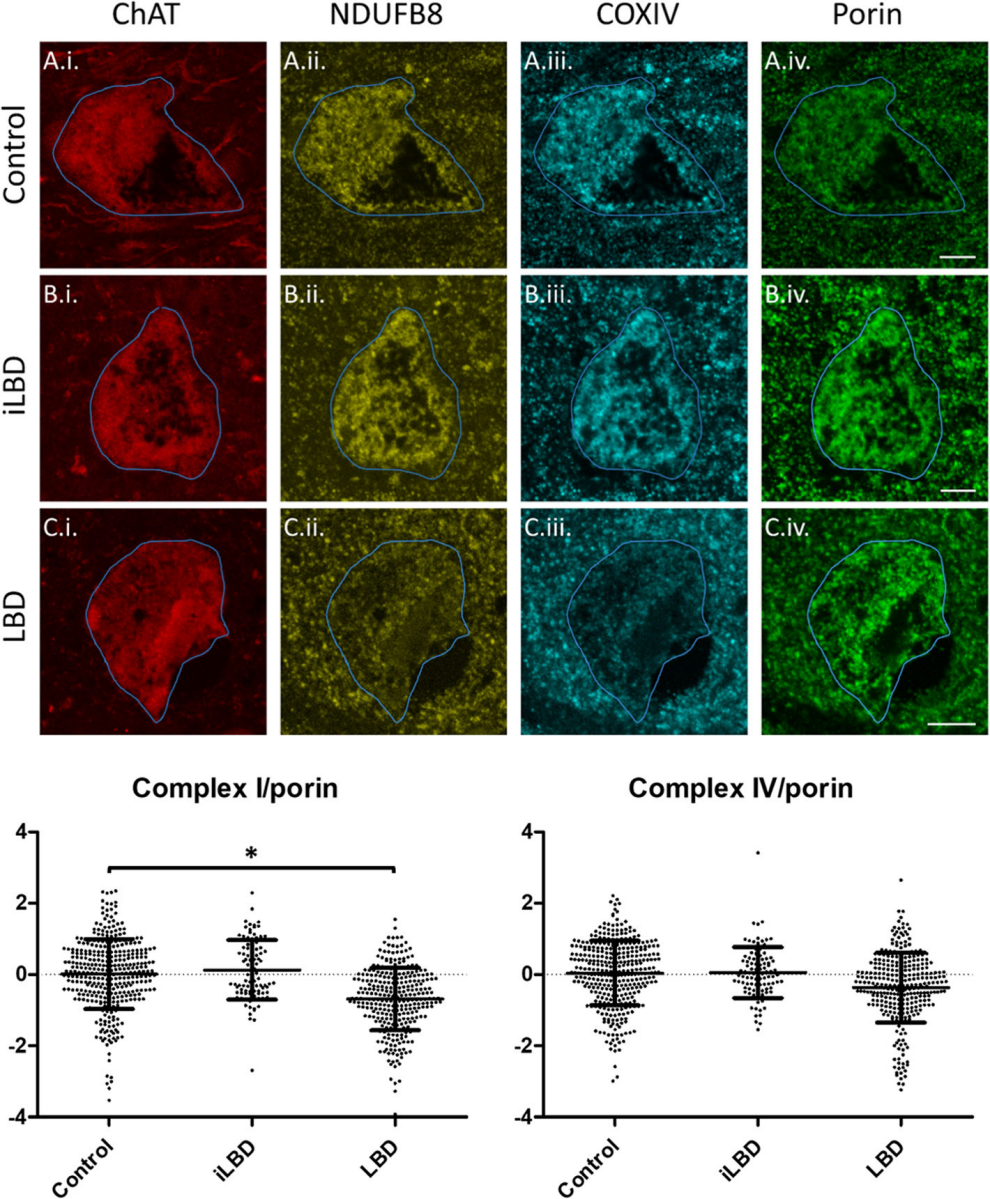
Photomicrographs demonstrating reductions in mitochondrial respiratory chain expression in nbM cholinergic neurons in LBD. Representative images demonstrating respiratory chain subunit expression in control (A.i.-A.iv.), iLBD (B.i.-B.iv.) and LBD (C.i.-C.iv.) cases. Scale bars = 10 μm. Dot plots show group level analysis of z scores of Complex I/NDUFB8 and IV/COXIV normalised to porin. Bars are means and standard deviation. *p < 0.05

Immunofluorescent analysis of the expression of the Complex I marker NDUFB8 and the Complex IV marker COXIV were conducted relative to the mitochondrial membrane marker porin in single nbM neurons to determine whether there were specific deficits in respiratory chain complexes relative to total mitochondrial mass (Fig. 2). Initial analysis of porin expression suggested it was significantly increased in iLBD and LBD cases compared to control (Supplementary Figure 1). Pooled data of cells per case per experimental group were evaluated in a mixed effects model to control for the effect of the pooled cells being obtained from eight individual cases. This analysis demonstrated that the LBD group was significantly reduced in Complex I relative to porin compared to the control group (− 0.75, *p* = 0.049), whilst the iLBD group was not significantly different from the control group (0.18, *p* = 0.749). In contrast, Complex IV relative to porin was not significantly different from control in LBD (− 0.40, *p* = 0.319) or iLBD (0.11, *p* = 0.854; Fig. 2), once variations between individuals were accounted for (Supplementary Figures 2 & 3).

### Reductions in respiratory chain markers vary across LBD cases

When individual neurons were stratified based on the degree of their deficiency (based on standard deviations from the control mean), LBD cases were highly variably affected by Complex I reductions (Supplementary Figure 4).

We asked whether variation in the proportion of cells with reduced Complex I expression (i.e. the proportion of cholinergic neurons whose z score was more than one standard deviation below the mean, as established from the control z distribution) was associated with cell loss, which is known to occur in the nbM in LBD and is thought to underlie cognitive dysfunction (4). Quantifying cell numbers in the nbM is difficult due to the lack of clear borders and anatomical differences along the extent of the nbM coupled with the difficulty in acquiring sections from precisely the same coronal level. Nevertheless, as has been conducted in previous studies [1], we counted the number of cholinergic neurons in the area of the nbM on a single 7 μm section stained with ChAT, and observed a trend towards a negative correlation between the number of cholinergic neurons and proportion of cells with reductions in Complex I (r_s_ = − 0.576, *p* = 0.088; Supplementary Figure 5) but not Complex IV (*p* = 0.204).

### Complex I reductions are greater in cells without Lewy bodies

**Fig. 3:**
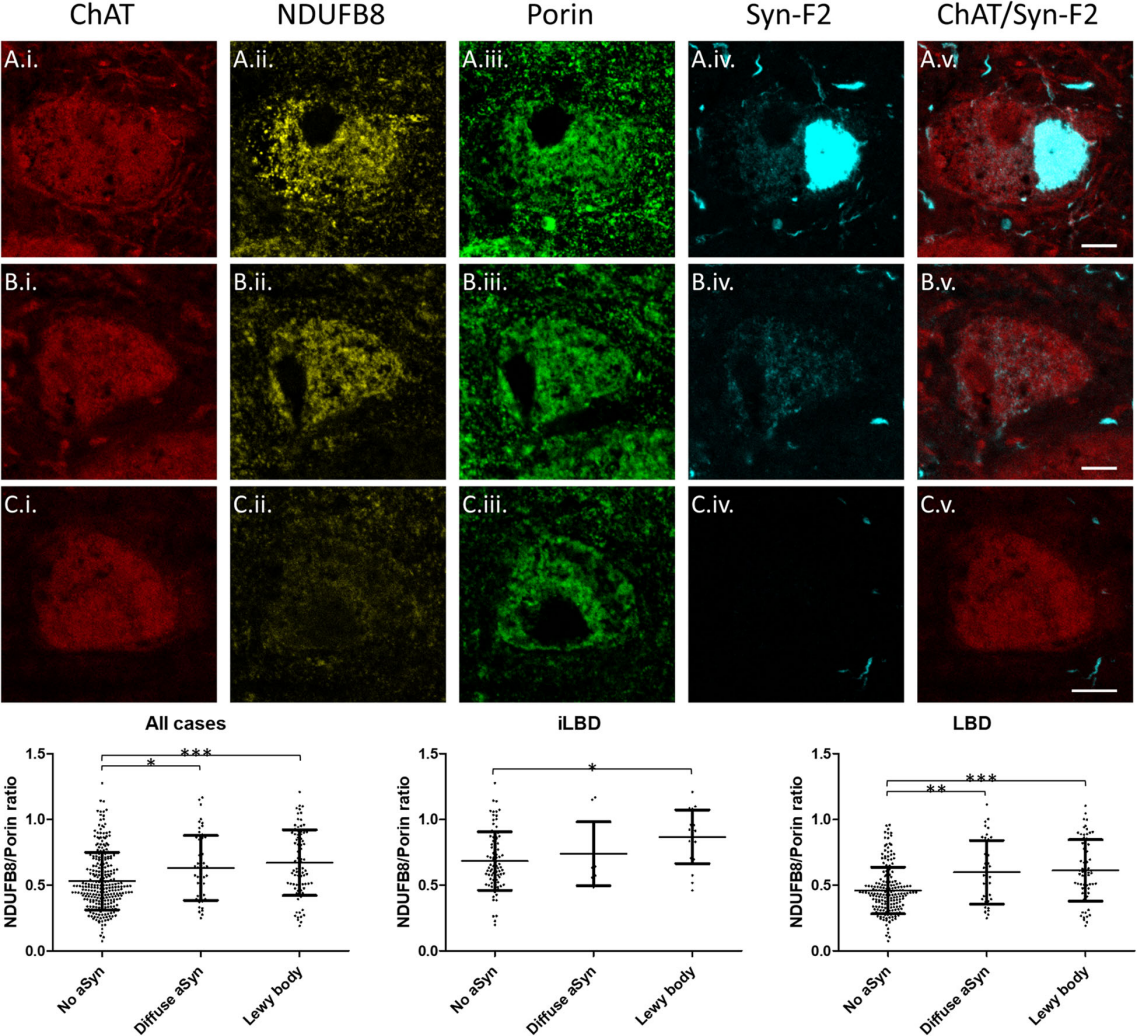
Photomicrographs demonstrating analysis of the Complex I marker NDUFB8 in nbM cholinergic neurons in LBD with or without the α-synuclein marker Syn-F2. Representative images demonstrating a cell with a Lewy body (A.i.-A.v.) and a cell with diffuse α-synuclein accumulation (B.i.-B.v.) with obvious Complex I/NDUFB8 expression, in contrast to a Complex I/NDUFB8 deficient cell with no α-synuclein immunoreactivity (C.i.-C.v.). Scale bars = 10 μm. Analysis of Complex I/NDUFB8 normalised to porin in cells without α-synuclein aggregates, cells with diffuse α-synuclein accumulations, and cells with Lewy bodies. Bars are means and standard deviation. **p < 0.01, ***p < 0.001

We next aimed to determine the relationship between Lewy body pathology and the observed reductions in Complex I expression. Initial observation of sections stained with the Syn-F2 antibody demonstrated two broad classes of α-synuclein aggregate formation: well-defined Lewy body-like structures and diffuse accumulations (Fig. 2). Therefore, we compared Complex I (NDUFB8) expression in cells without α-synuclein aggregates to those with Lewy bodies or diffuse aggregates. Due to a lack of suitable tissue, one case was excluded from this analysis (LBD 8). The Complex I marker NDUFB8 was normalised to porin and evaluated in cells without any α-synuclein aggregates compared to cells with diffuse accumulations and those with Lewy bodies. Analysis across all cases with Lewy bodies in the nbM demonstrated a significant main effect (K-W χ^2^ = 26.29, *p* < 0.0001), with post-hoc analyses demonstrating higher expression of Complex I in cells with Lewy bodies (*N* = 83; *p* < 0.001) and cells with diffuse α-synuclein aggregates (*N* = 51; *p* < 0.01) compared to cells without any α-synuclein aggregates (*N* = 272). However, there was no significant difference between cells with Lewy bodies and cells with diffuse α-synuclein aggregates (all data summarised in Fig. 3). There was no difference in porin levels between cells with and without α-synuclein aggregates. When data was broken down into individual cases, the same general trend of higher Complex I expression in cells with Lewy bodies was observed within individual cases (Supplementary Figure 6).

We also determined NDUFB8 expression relative to porin in cells with and without α-synuclein aggregates between groups with Lewy body pathology (i.e. LBD and iLBD). LBD cases had a significant main effect (K-W χ^2^ = 29.04, *p* < 0.0001), with post-hoc analyses demonstrating higher expression of NDUFB8 relative to porin in cells with Lewy bodies (*N* = 64; *p* < 0.001) and cells with diffuse α-synuclein accumulation (*N* = 39; *p* < 0.001) compared to cells without any α-synuclein aggregates (*N* = 187). iLBD cases also had a significant main effect (K-W χ^2^ = 9.32, *p* < 0.01), with post-hoc analyses demonstrating higher expression of NDUFB8 relative to porin in cells with Lewy bodies (*N* = 19; *p* < 0.001) compared to cells without any α-synuclein aggregates (*N* = 85). There was no significant difference in NDUFB8 relative to porin between cells with diffuse α-synuclein accumulations (*N* = 12) and cells with or without Lewy bodies. All data is summarised in Fig. 3.

We noted that porin and NDUFB8 were present within Lewy bodies, whether viewed as flat 2D images or following z stack. To ensure mitochondria within Lewy bodies were not artefactually altering expression of mitochondrial proteins within cells with Lewy bodies we also conducted this analysis having first subtracted the area occupied by Lewy bodies from every channel. However, this analysis demonstrated a similar trend, with a significant main effect when data were pooled (K-W χ^2^ = 18.35, *p* < 0.001) and cells without α-synuclein containing significantly lower NDUFB8 relative to porin than cells with Lewy bodies (p < 0.001) and cells with diffuse α-synuclein (*p* < 0.05). Cells with Lewy bodies also had higher levels of NDUFB8 relative to porin when only cases with LBD (*p* < 0.001) or iLBD (*p* < 0.05) were analysed (Supplementary Figure 7).

### Respiratory chain deficiencies are unrelated to neurodegenerative pathology burden

Correlational analyses between α-synuclein percentage area stained per case and the percentage of cells with reduced respiratory chain subunit expression (i.e. more than one standard deviation below the control mean) demonstrated a negative correlation between burden of α-synuclein pathology and percentage of cells with reductions in Complex I (r_s_ = − 0.900, *p* = 0.002) and Complex IV (r_s_ = − 0.814, *p* = 0.011; Supplementary Figure 8). These data suggest that lower levels of α-synuclein pathology are associated with higher levels of Complex I deficiency. We suspected these data may be driven by the inherent bias of measures of percentage area immunoreactive for α-synuclein towards the number of large Lewy bodies, thus biasing such measures towards cases with higher numbers of cells. Therefore, we correlated the percentage of total cells bearing Lewy bodies with the percentage of cells with reduced levels of respiratory chain subunit expression per case. This analysis demonstrated no relationship between the percentage of cells bearing Lewy bodies and the percentage of cells deficient in Complex I (*p* = 0.581) or Complex IV (*p* = 0.643; Supplementary Figure 8). There was also no relationship between percentage of cells bearing Lewy bodies and cell density (*p* = 0.437; Supplementary Figure 9). There was no significant relationship between the proportion of Complex I deficient neurons and tau (*p* = 0.233) or amyloid-β (*p* = 0.982).

## Discussion

The present study has demonstrated that the nbM, a critical region for cortical cholinergic innervation, evidences reductions in Complex I of the mitochondrial respiratory chain in LBD that may relate to neuronal loss. Notably, Complex I reductions were not observed in asymptomatic iLBD cases, despite these cases manifesting Lewy bodies of similar abundance to clinically symptomatic cases of LBD in this region. Furthermore, cells with Lewy bodies were found to have higher expression levels of Complex I than cells without Lewy bodies, with the most severe deficiencies observed in cells without Lewy bodies. Taken together, these findings suggest that the nbM is subject to reductions in Complex I expression in symptomatic LBD but that Lewy body formation in neurons may be protective against these changes.

LBD are notable amongst neurodegenerative disorders as neuronal loss is not as uniform as that observed in Alzheimer’s disease [7, 9], nor is every region that manifests Lewy bodies subject to cell loss [2, 6]. It has been suggested that regions with particularly high energy demands would render some cellular populations particularly vulnerable to degeneration, secondary to mitochondrial deficits [39]. Therefore, the present study reporting mitochondrial respiratory chain deficits in the nbM, a region whose cells have high energy demands and are also susceptible to Lewy body pathology and significant cell loss [8], is consistent with the hypothesis that regions with high energy demands are vulnerable to neuronal loss and mitochondrial dysfunction in LBD.

Consistent with the hypothesis that regions with high energy demands may be vulnerable to neurodegeneration in the context of mitochondrial dysfunction in LBD, we noted a statistically non-significant trend towards a correlation between reductions in Complex I and cell loss. However, it should be noted that our evaluation of cell number, whilst necessary given the aforementioned issues in quantifying cell density in the nbM, is less likely to provide data as robust as stereological estimation based on sampling of the entire structure [19]. Although our estimates of cholinergic neuron number are similar to those previously reported in the nbM in LBD with the same method [1], it is impossible to exclude the possibility that differences in the area sampled across cases influenced cell density, and this could be contributing to the trend towards a relationship between reductions in Complex I and cell loss. Future studies are necessary to better understand relationships between reductions in Complex I and cell loss in LBD. Nevertheless, the underlying cause of reduced expression of respiratory chain subunits in the nbM in LBD is not clear, but α-synuclein has been demonstrated to interact with mitochondria [22, 42], and reductions in Complex I expression have been reported in the substantia nigra, where it was related to mtDNA deletions, and cortex in LBD *post-mortem* tissue [12, 34, 36].

The aggregation of α-synuclein into Lewy bodies is thought to be a central event in LBD, and the formation of Lewy bodies is necessary for a neuropathological diagnosis of LBD [5, 24]. Previous studies have suggested that the formation of Lewy bodies is cytotoxic to neurons due to the propensity of α-synuclein aggregates to sequester and accumulate critical cellular organelles, particularly mitochondria [21, 32]. However, the present study challenges the canonical view that Lewy bodies induce mitochondrial dysfunction by demonstrating, within nbM neurons, that Lewy bodies are associated with higher levels of Complex I relative to total mitochondrial mass. The observation that Lewy bodies are associated with higher expression of Complex I markers conflicts with the results of previous studies that interpreted the presence of mitochondria within Lewy bodies as a cytotoxic event [21, 32], but are consistent with studies demonstrating Lewy body-bearing neurons are less likely to manifest Complex I deficiency in the substantia nigra [12, 34]. Notably, we have extended previous findings in the substantia nigra by demonstrating higher expression of Complex I in cells with Lewy bodies in the nbM, suggesting this may be a common feature of Lewy body bearing neurons.

In the present study, iLBD cases had no respiratory chain dysfunction despite a comparable level of Lewy body pathology in the nbM, suggesting that respiratory chain dysfunction is a key distinction between symptomatic and asymptomatic cases. It is not clear why mitochondrial respiratory chain complexes would differ between LBD and iLBD cases in the context of similar local neurodegenerative pathology burdens. ChAT catalyses the production of acetylcholine from acetyl-CoA, a key enzyme for mitochondrial energy production [38] and, as ChAT expression is thought to be proportionate to the rate of acetylcholine synthesis and substrate availability [38], one could speculate that the observed elevations in ChAT in the iLBD group reflect higher levels of acetylcholine and acetyl-CoA. Elevations in acetylcholine synthesis and acetyl-CoA expression in iLBD cases could be a plausible explanation for preserved respiratory chain expression and cognitive capacity in iLBD cases, given that the abundance of acetyl-CoA reflects the general energetic state of a cell [31] and acetylcholine has critical roles in higher cognitive processes that are compromised in LBD [8]. However, it should be emphasised that it is difficult to draw strong conclusions when the iLBD cohort only comprised two patients. Limited numbers of iLBD cases were due to the rarity of cases with widespread Lewy body pathology and an absence of clinical features, and we acknowledge this as a limitation of the present study.

The reasons for the association between the presence of a Lewy body and greater cellular expression of Complex I are unclear, but there are a number of plausible explanations. It has been reported that Lewy bodies contain dystrophic mitochondria [37] and, therefore, one could speculate that Lewy bodies accumulate mitochondria in response to mitophagy deficits [3], resulting in less respiratory chain deficiencies in neurons harbouring Lewy bodies. Mitophagy deficits may be a plausible explanation given the significant increases in porin we observed in LBD and iLBD cases though it is also possible that the observed increase in porin expression reflects oligomerisation into large channels that facilitate release of pro-apoptotic proteins [38]. It is also possible that Lewy bodies accumulate and compartmentalise more toxic species of α-synuclein, such as putatively toxic oligomers [11, 27]. In this regard, cells containing Lewy bodies would be expected to have less respiratory chain deficiencies than those containing more toxic species, which may be too small to be visualised by light microscopy. Finally, it is possible that the generation of ATP by mitochondria is necessary for the aggregation of α-synuclein into Lewy bodies, and therefore cells with greater expression of respiratory chain complexes are more likely to harbour Lewy bodies. However, given the propensity of α-synuclein to aggregate in the absence of ATP in vitro this seems less likely [29], but it may be the case that formation of the structure of Lewy bodies, reminiscent of an aggresome response, is dependent on ATP. It should be noted that none of these explanations are mutually exclusive.

It is important to emphasise that mitochondrial dysfunction is not the only proposed mechanism underlying cellular dysfunction in LBD, so it is possible that Lewy bodies could be protective against reductions in markers of the mitochondrial respiratory chain but cause cellular impairment through other pathways, such as disruption of the microtubule network or DNA damage [32]. It is also important to note that the present findings or interpretation do not necessarily diminish the central role ascribed to α-synuclein in LBD. For example, it is possible that increased expression of α-synuclein impairs the autophagy-lysosomal system, inducing mitophagy impairments thought to contribute to accumulation of damaged mitochondria, and formation of Lewy bodies in some cells to ameliorate the cytotoxic effect of this accumulation. Nevertheless, further research is warranted to determine how Lewy body accumulation contributes to mitochondrial function and cellular health in LBD.

In summary, the present study reports reduction in Complex I of the mitochondrial respiratory chain in nbM neurons in LBD compared to control and iLBD cases, and this may be related to neuronal loss in this region. However, contrary to recent findings from in vitro studies, cells with Lewy bodies are associated with higher proportions of mitochondria with intact respiratory chain subunits compared to neurons without Lewy bodies. We speculate these findings suggest Lewy bodies perform a protective function, perhaps by accumulating more toxic species of α-synuclein and/or damaged mitochondria, thus protecting the cell from the deleterious effects of accumulated cytotoxic components. However, further studies are warranted to determine whether Lewy bodies are a protective phenomenon, or whether they exert cytotoxic effects through pathways other than mitochondrial dysfunction.

## Conclusion

The present study has demonstrated that reductions in Complex I of the mitochondrial respiratory chain are observed in the nbM of individuals with LBD, and these may relate to the neuronal loss observed in this region that is thought to underlie cognitive deficits. Notably, such reductions were not observed in individuals with iLBD, who had Lewy body pathology but no clinical symptoms, suggesting mitochondrial damage may relate more closely to phenotype than the presence of absence of Lewy bodies. Contrary to the view that Lewy bodies induce cellular dysfunction underlying clinical symptomatology in LBD, cells with Lewy bodies had less dysfunctional mitochondria than proximal neurons without Lewy bodies, suggesting Lewy bodies may have a protective role in LBD.

## Supplementary information

**Additional file 1: Supplementary Figure 1.** ChAT expression is elevated in iLBD and porin is increased in iLBD and LBD. **Supplementary Figure 2.** Merged images of respiratory chain subunits demonstrate co-localisation of mitochondrial markers within ChAT-immunoreactive neurons. **Supplementary Figure 3.** Complex I and IV expression relative to porin in individual LBD cases are demonstrated relative to control and iLBD cases. **Supplementary Figure 4.** Data across individual LBD cases demonstrated highly variable patterns of deficiency across cases. **Supplementary Figure 5.** Scatterplot demonstrating the relationship between total number of ChAT+ neurons per case and percentage of cells with Complex I expression <1 standard deviation between the control group mean. **Supplementary Figure 6.** When NDUFB8 relative to porin was plotted across individual cells per LBD case we observed a general trend of cells with no α-synuclein having lower levels compared to cells with Lewy bodies. **Supplementary Figure 7.** Lewy body bearing neurons had higher levels of NDUFB8 relative to porin when the area occupied by the Lewy body was removed from the analysis. **Supplementary Figure 8.** Correlational analyses of percentage area occupied by α-synuclein and respiratory chain deficiencies. **Supplementary Figure 9.** Correlational analysis of cell count and percentage of cells bearing Lewy bodies.

## Data Availability

The data that support the findings of this study are available from the corresponding author upon reasonable request.

## Abbreviations

ATP: Adenosine triphosphate
ChAT: Choline acetyltransferase
COX4: Cytochrome oxidase subunit 4
EDTA: Ethylenediaminetetraacetic acid
HRP: Horseradish peroxidase
IgG: Immunoglobulin G
iLBD: Incidental Lewy body disease
K-W: Kruskal-Wallis
LBD: Lewy body dementia
NBTR: Newcastle Brain Tissue Resource
NDUFB8: NADH dehydrogenase [ubiquinone] 1 beta subcomplex unit 8
nbM: Nucleus basalis of Meynert
OD: Optical density
TBST: Tris-buffered saline with Tween20
VDAC1: Voltage-dependent anion channel 1
