# Supplementary material for: Complex I reductions in the nucleus basalis of Meynert in Lewy body dementia: the role of Lewy bodies
Source: Acta Neuropathol Commun. 2020 Jul 9;8:103. doi: 10.1186/s40478-020-00985-8 (PMC7346628; doi:10.1186/s40478-020-00985-8)
Supplement: Supplementary file 1 — Additional file 1: Supplementary Figure 1. ChAT expression is elevated in iLBD and porin is increased in iLBD and LBD. Supplementary Figure 2. Merged images of respiratory chain subunits demonstrate co-localisation of mitochondrial markers within ChAT-immunoreactive neurons. Supplementary Figure 3. Complex I and IV expression relative to porin in individual LBD cases are demonstrated relative to control and iLBD cases. Supplementary Figure 4. Data across individual LBD cases demonstrated highly variable patterns of deficiency across cases. Supplementary Figure 5. Scatterplot demonstrating the relationship between total number of ChAT+ neurons per case and percentage of cells with Complex I expression <1 standard deviation between the control group mean. Supplementary Figure 6. When NDUFB8 relative to porin was plotted across individual cells per LBD case we observed a general trend of cells with no α-synuclein having lower levels compared to cells with Lewy bodies. Supplementary Figure 7. Lewy body bearing neurons had higher levels of NDUFB8 relative to porin when the area occupied by the Lewy body was removed from the analysis. Supplementary Figure 8. Correlational analyses of percentage area occupied by α-synuclein and respiratory chain deficiencies. Supplementary Figure 9. Correlational analysis of cell count and percentage of cells bearing Lewy bodies. [file 40478_2020_985_MOESM1_ESM.docx]

SUPPLEMENTARY FIGURES

*
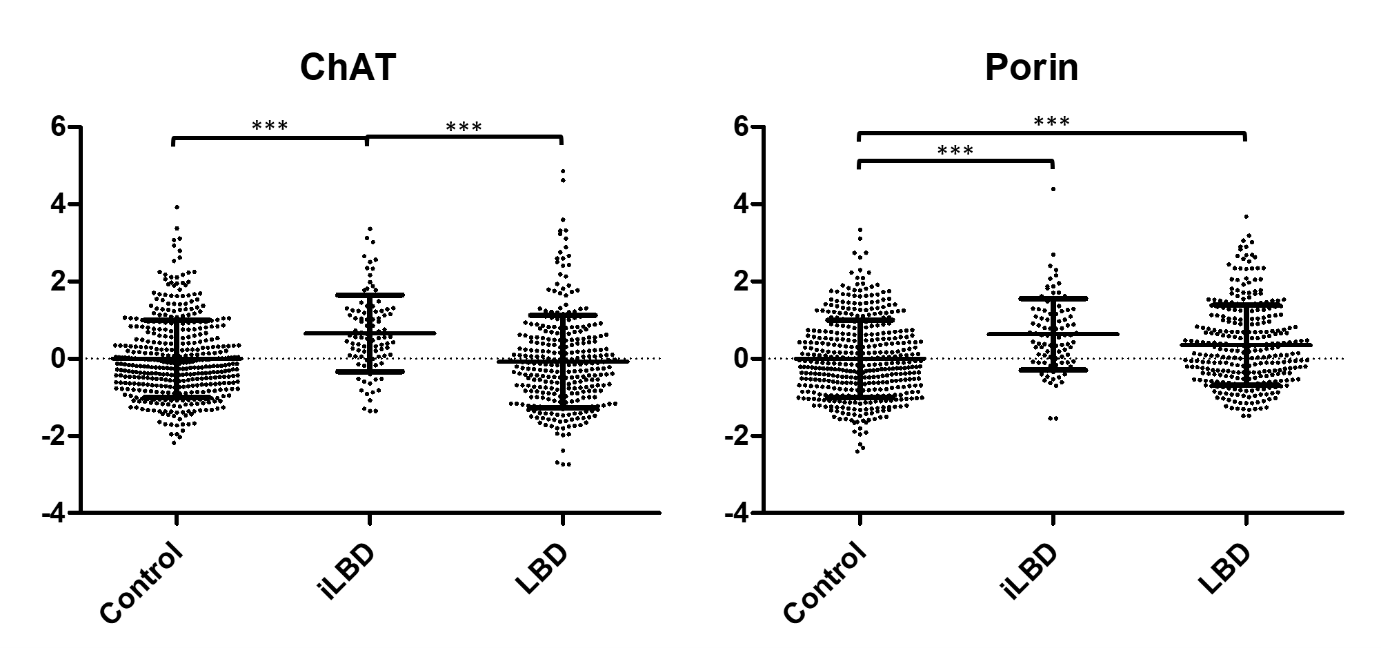
*

*Supplementary Figure 1: ChAT expression is elevated in iLBD and porin is increased in iLBD and LBD. Immunofluorescent analysis of the intensity of the cholinergic neuron marker ChAT in individual neurons demonstrated a significant main effect across groups (K-W χ^2^=42.50, p<0.0001). Post-hoc analysis using Dunn’s test for multiple comparisons demonstrated significant increases in expression of ChAT in iLBD when compared to control (p<0.001) and LBD (p<0.001).* *There were no significant differences in cellular ChAT expression between control and LBD. Immunofluorescent analysis of the intensity of the mitochondrial outer membrane marker porin/VDAC1, as a marker of total mitochondrial mass, demonstrated a significant main effect across groups (K-W χ^2^=41.29, p<0.0001). Post-hoc analysis using Dunn’s test for multiple comparisons demonstrated significant increases in expression of porin in iLBD (p<0.001) and LBD (p<0.001) compared to control.*

*
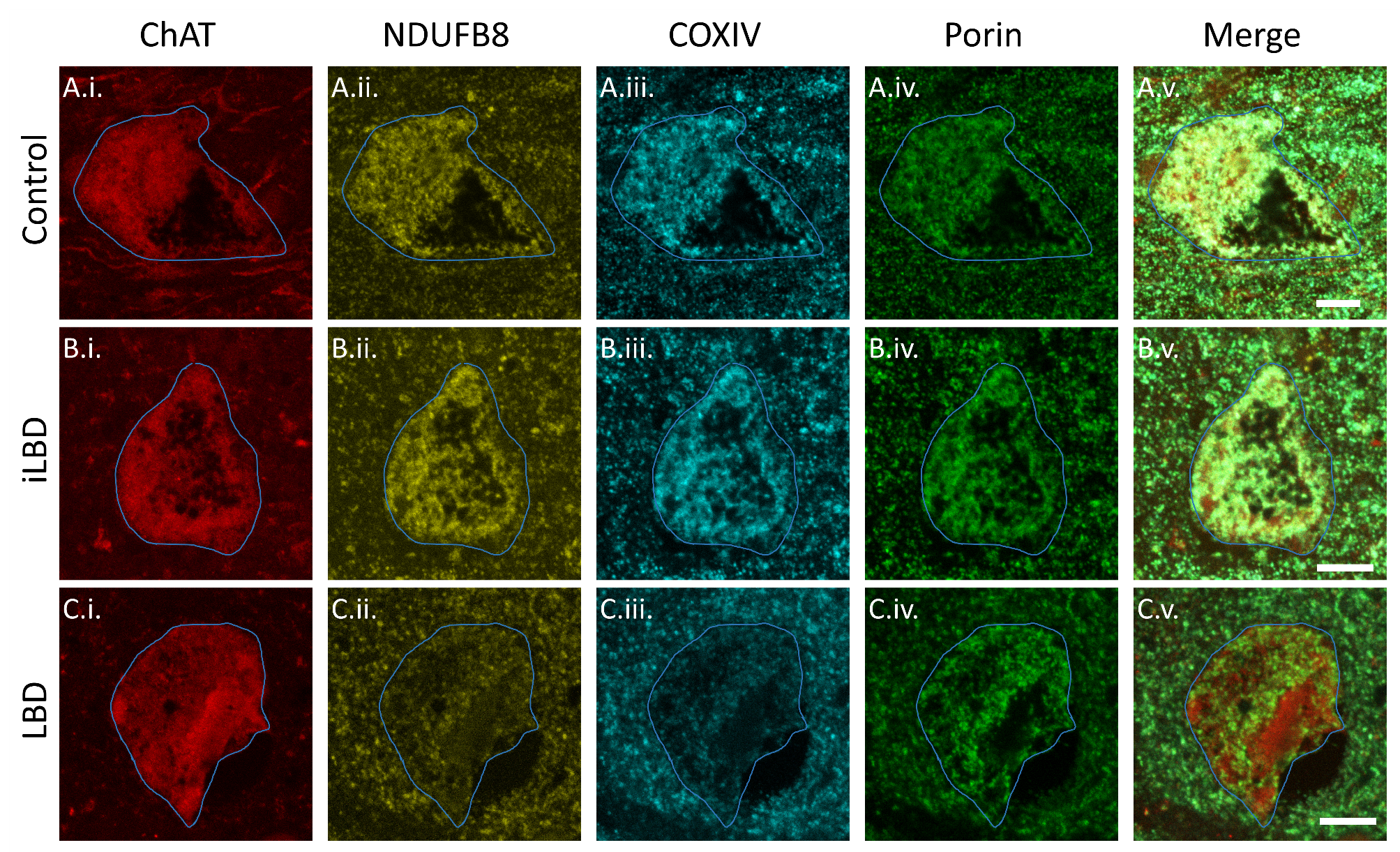
*

*Supplementary Figure 2: Merged images of respiratory chain subunits demonstrate co-localisation of mitochondrial markers within ChAT-immunoreactive neurons. Scale bars = 10 µm.*

*
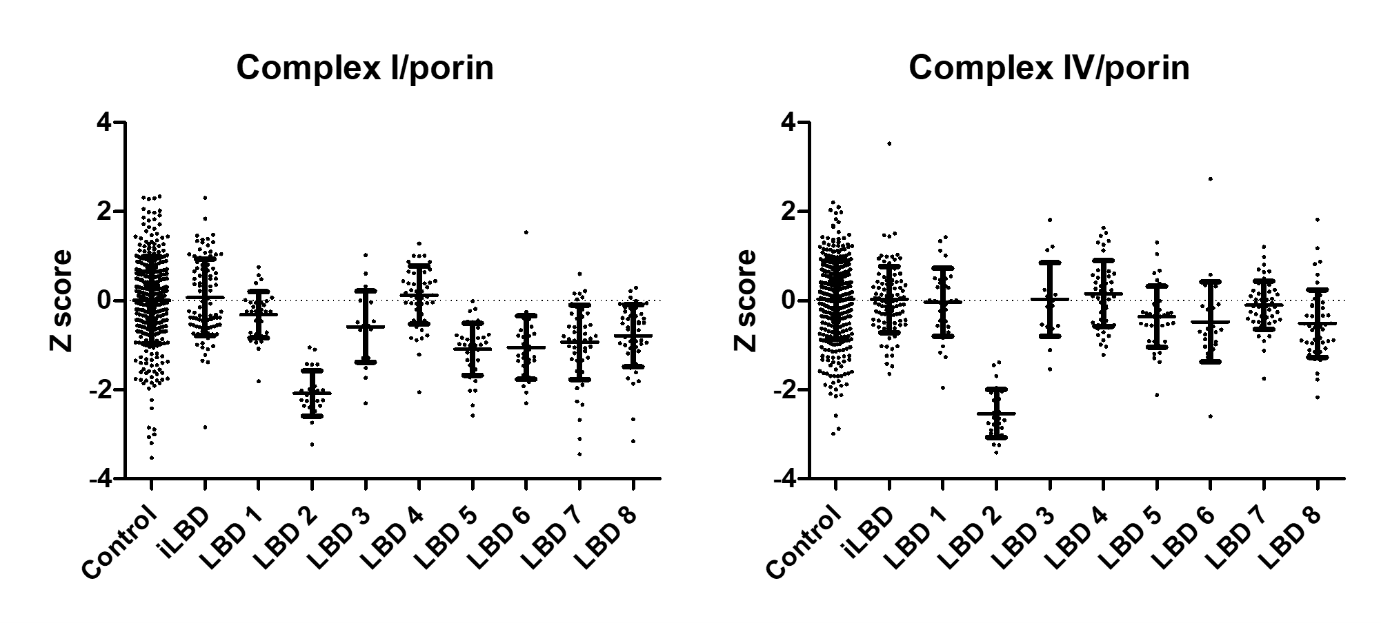
*

*Supplementary Figure 3: Complex I and IV expression relative to porin in individual LBD cases are demonstrated relative to control and iLBD cases, highlighting substantial variability.*

*
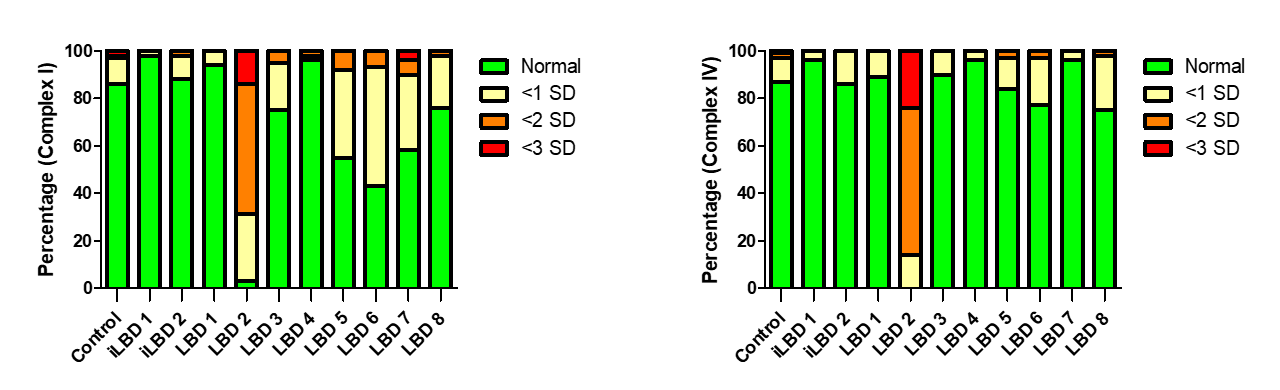
*

*Supplementary Figure 4: Data across individual LBD cases demonstrated highly variable patterns of deficiency across cases, with one case (LBD case 2) highly deficient, in comparison to other LBD cases, some of which showed remarkable preservation (LBD cases 1 and 4). Note the LBD group were still significantly reduced in Complex I and IV when LBD case 2 is removed from the analysis.*

*Supplementary Figure 5: Scatterplot demonstrating the relationship between total number of ChAT+ neurons per case and percentage of cells with Complex I expression <1 standard deviation between the control group mean (r_s_=-0.58, p=0.0883).*

*
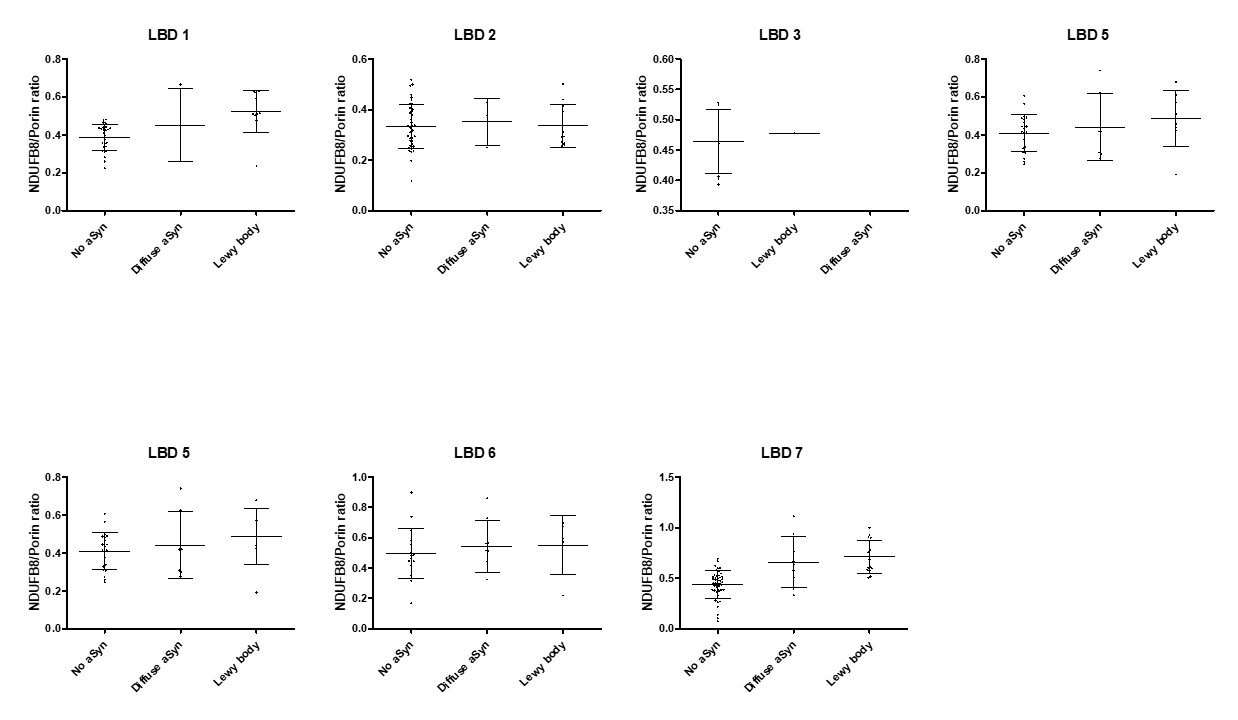
*

*Supplementary Figure 6: When NDUFB8 relative to porin was plotted across individual cells per LBD case we observed a general trend of cells with no α-synuclein having lower levels compared to cells with Lewy bodies.*

*
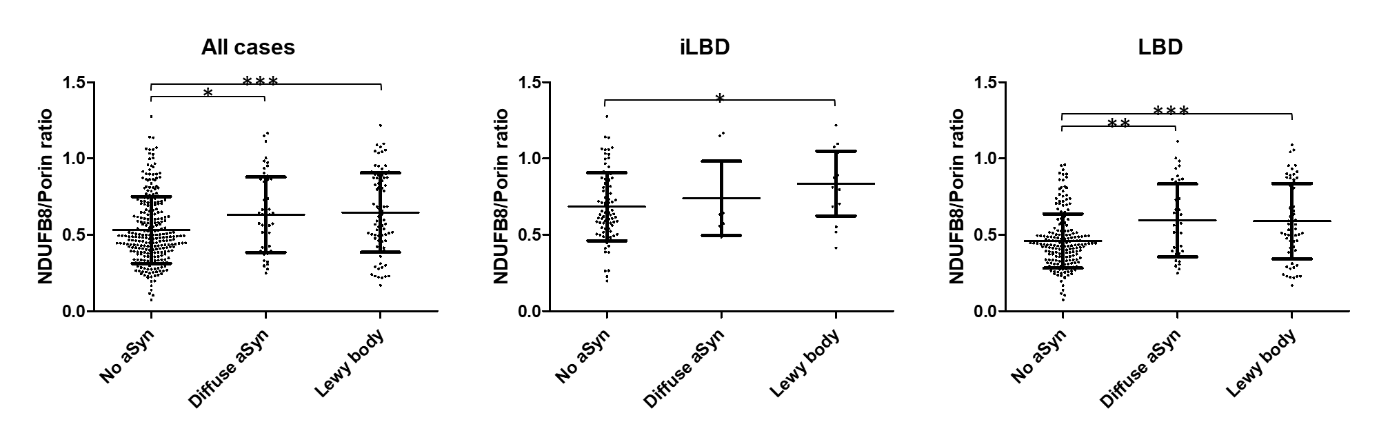
*

*Supplementary Figure 7: Lewy body bearing neurons had higher levels of NDUFB8 relative to porin when the area occupied by the Lewy body was removed from the analysis. *p<0.01, **p<0.001, ***p<0.0001.*

*Supplementary Figure 8: Correlational analyses of percentage area occupied by α-synuclein and respiratory chain deficiencies demonstrate significant negative correlations between α-synuclein percentage area and percentage of Complex I* *(*r_s_=-0.900, p=0.002*) and Complex IV (*r_s_=-0.814, p=0.011*) deficient cells. However, percentage of cells bearing Lewy bodies did not demonstrate significant correlations with percentage of cells Complex I (r_s_=-0.217, p=0.581) or Complex IV (r_s_=-0.187, p=0.643).*

*
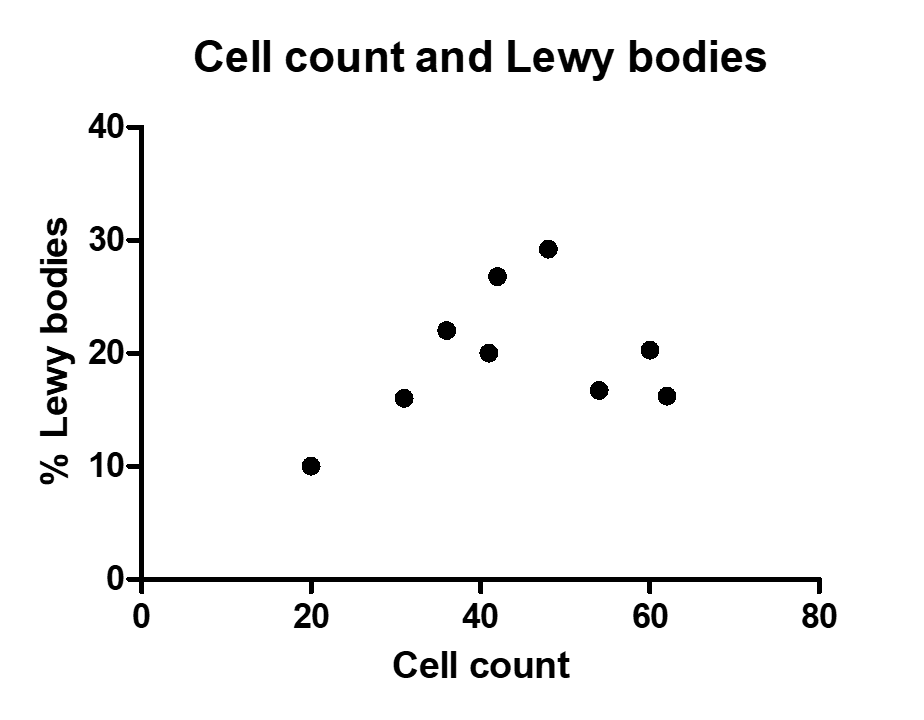
*

*Supplementary Figure 9: Correlational analysis of cell count and percentage of cells bearing Lewy bodies demonstrated no significant correlation (r_s_=0.3000, p=0.4366).*
